# Supplementary material for: A novel mechanism for bacterial sporulation based on programmed peptidoglycan degradation
Source: bioRxiv. 2025 Jun 26:2025.06.26.661752. Preprint. [Version 1] doi: 10.1101/2025.06.26.661752 (PMC12265050; doi:10.1101/2025.06.26.661752)
Supplement: Supplement 1 [file media-1.pdf]

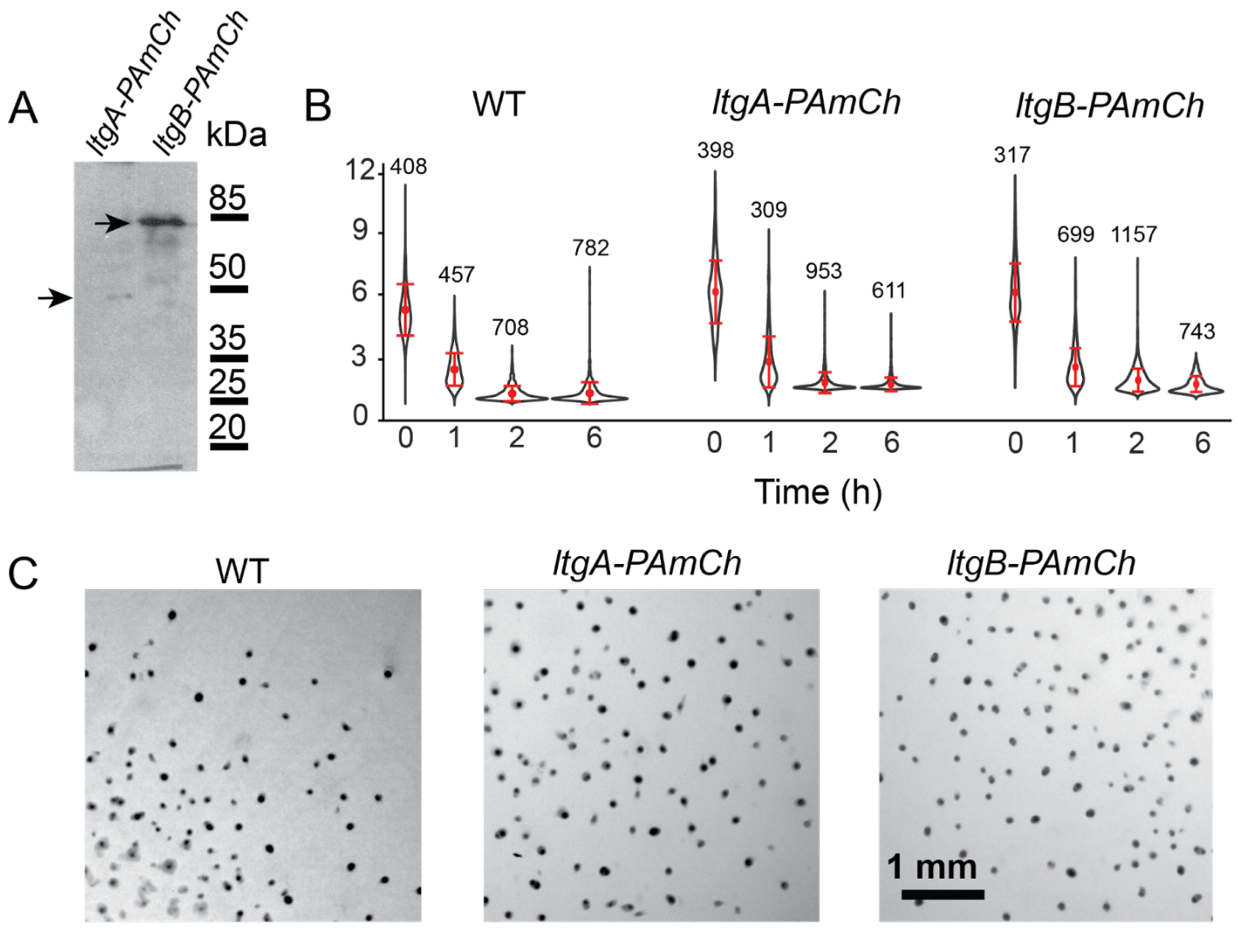

Fig. S1. PAmCherry-labeled LtgA and LtgB are fully functional. A) PAmCherry-labeled LtgA and LtgB are expressed as full-length proteins, which was detected by an anti-mCherry antibody. **B)** The PAmCherry labels do not affect glycerol-induced sporulation. Here and in Fig. S2, quantitative analysis of glycerol-induced sporulation using the length/width ratio (L/W) of cells. Whiskers indicate the 25th - 75th percentiles and red dots the median. The total number of cells analyzed is shown on top of each plot. **C**) The PAmCherry labels do not affect starvation-induced sporulation on agar surfaces.

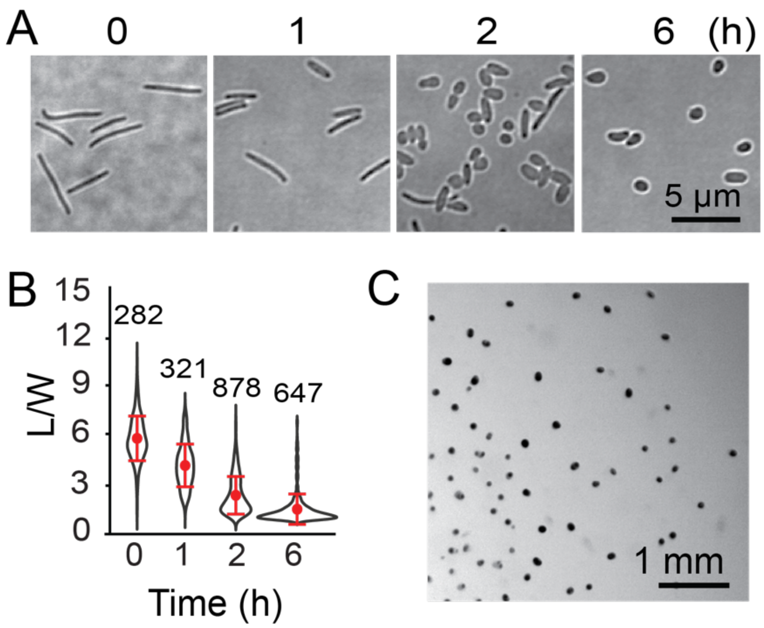

Fig. S2. Cells carrying *P_{van}-murA* grown in the absence of vanillate form spores normally through both sporulation pathways. The leak expression of MurA by the vanillate-inducible promoter does not affect glycerol-induced sporulation (**A, B)** or on fruiting body formation (**C**).
